# Third national biobank for population-based seroprevalence studies in the Netherlands, including the Caribbean Netherlands

**DOI:** 10.1186/s12879-019-4019-y

**Published:** 2019-05-28

**Authors:** Janneke Doortje Maria Verberk, Regnerus Albertus Vos, Liesbeth Mollema, Jeffrey van Vliet, Joanna Wilhelmina Maria van Weert, Hester Ellen de Melker, Fiona Regina Maria van der Klis

**Affiliations:** Centre for Infectious Disease Control, National Institute for Public Health and the Environment (RIVM), Bilthoven, the Netherlands

**Keywords:** Serosurveillance, Immunisation, Biobank, Study design, The Netherlands, Caribbean Netherlands

## Abstract

**Background:**

This paper outlines the methodology, study population and response rate of a third large Dutch population-based cross-sectional serosurvey carried-out in 2016/2017, primarily aiming to obtain insight into age-specific seroprevalence of vaccine-preventable diseases to evaluate the National Immunization Programme (NIP). In addition, Caribbean Netherlands (CN) was included, which enables additional research into tropical pathogens.

**Methods:**

A two-stage cluster sampling technique was used to draw a sample of Dutch residents (0–89 years) (NS), including an oversampling of non-Western migrants, persons living in low vaccination coverage (LVC) areas, and an extra sample of persons born in Suriname, Aruba and the former Dutch Antilles (SAN). A separate sample was drawn for each Caribbean island. At the consultation hours, questionnaires, blood samples, oro- and nasopharyngeal swabs, faeces, − and only in the Netherlands (NL) saliva and a diary about contact patterns – were obtained from participants. Vaccination- and medical history was retrieved, and in CN anthropometric measurements were taken.

**Results:**

In total, blood samples and questionnaires were collected from 9415 persons: 5745 (14.4%) in the NS (including the non-Western migrants), 1354 (19.8%) in LVC areas, 501 (6.9%) SAN, and 1815 (23.4%) in CN.

**Conclusions:**

This study will give insight into protection of the population against infectious diseases included in the NIP. Research based on this large biobank will contribute to public health (policy) in NL and CN, e.g., regarding outbreak management and emerging pathogens. Further, we will be able to extend our knowledge on infectious diseases and its changing dynamics by linking serological data to results from additional materials collected, environmental- and pharmacological data.

**Electronic supplementary material:**

The online version of this article (10.1186/s12879-019-4019-y) contains supplementary material, which is available to authorized users.

## Background

The seroprevalence of National Immunization Programme (NIP)-targeted diseases is periodically monitored in the Netherlands (NL) by national seroepidemiological (PIENTER) studies, executed by the National Institute for Public Health and the Environment (RIVM) in collaboration with Public Health Services and municipalities. The first serosurvey was performed in 1995/1996 [1] and the second in 2006/2007 [2].

Gathering seroepidemiological data forms an important tool for the evaluation and optimization of the NIP, and gives insight into the protection against infectious diseases in different (sub)groups in the population. The results of previous Dutch serosurveys have contributed to vaccine policy, e.g., during the measles epidemic in 2013/2014, data on seroprevalence (particular the maternal antibody levels in infants) from the second Dutch serosurvey in 2006/2007 were used to advise on measles-mumps-rubella (MMR)-vaccination at an earlier age [3]; tetanus seroprevalence data led to an advice regarding a more restricted use of tetanus prophylaxis [4]; and the decision to revaccinate against meningococcal C disease at an adolescent age with a tetravalent vaccine were partly based on these data [5].

Since the last serosurvey in 2006/2007 several adaptations in de NIP were applied (Table 1) and some (small) outbreaks occurred, e.g., measles outbreak in the Dutch Bible Belt in 2013/2014 [6], mumps among vaccinated young adults [7] and increased incidence of meningococcal serogroup W disease since 2015 [8]. Events like these will have impact on the immune status in the population and justify close investigation. In addition, monitoring the seroprotection of the population is required at regular time intervals as vaccination can affect the dynamics of infectious diseases on the long term, for instance leading to an increasing age of infection or waning antibody levels, e.g., against diphtheria and measles [3, 9]. For these reasons, a third seroepidemiological study (PIENTER-3) was performed [10] to identify (new) population groups at risk for infectious diseases and to evaluate the adaptations made in the vaccination scheme in order to improve the overall quality of the programme.

**Table 1:** Adaptations in the National Immunization Programme (NIP) in the Netherlands from 2006 to 2018

Year	Vaccination	Adaptation in the Dutch NIP
2018	Meningococcal ACWY vaccination	Change from MenC conjugate vaccine administered at 14 months of age to MenACWY conjugate vaccine.
2014	Human Papillomavirus vaccination	Change from 3 vaccinations to 2 vaccinations administered at 12 years of age.
2013	Pneumococcal vaccination	Change from 4 vaccinations administrated at 2, 3, 4 and 11 months of age to 3 vaccinations at 2, 4 and 11 months.
2011	Hepatitis B vaccination	Change from vaccination offered to infants at risk to vaccination for all children administered at 2, 3, 4 and 11 months of age, via DTaP-IPV-Hib-HepB.
2011	Pneumococcal vaccination	Change from vaccination against 7 serotypes to vaccination against 10 serotypes.
2009	Human Papillomavirus vaccination	Introduction of vaccination for girls 12 years of age with catch-up for girls up to 17 years of age.
2006	Pneumococcal vaccination	Introduction of vaccination administered at 2, 3, 4 and 11 months of age, simultaneously with DTaP-IPV-Hib-(HepB).

This third study has been extended with the collection of saliva and faecal samples, as well as oro- and nasopharyngeal swabs, creating a more comprehensive biobank for the Dutch population. With the collection of these diverse human materials, accompanied by extensive individual information from questionnaires and the linkage with other data sources (medication histories and environmental exposures), this biobank harbours a wealth of information.

Importantly, this serosurvey has been expanded to include the Caribbean Netherlands (CN) for the first time (Health Study Caribbean Netherlands, HSCN). The Caribbean islands Bonaire, St. Eustatius and Saba (together CN) are officially part of NL and considered public entities under Dutch law since October 10, 2010. Hence, public health falls under the direct responsibility of the Dutch government, e.g., the supply, execution and monitoring of the NIP. Strikingly, no data on protection against infectious diseases and associated risk factors are available. The need for knowledge is underlined by outbreaks of measles and diphtheria in neighboring countries in Latin America [11, 12] and epidemics of vector-borne diseases in the Caribbean region (e.g., zika, dengue and chikungunya) [13–15].

This paper outlines the design of population-based cross-sectional serosurveys in NL and CN, its study population and response rates. Subsequently, future research possibilities of this extensive data collection and experience with conducting large population-based public health research in CN will be described.

## Methods

### Study population and sample design

**Fig. 1:**
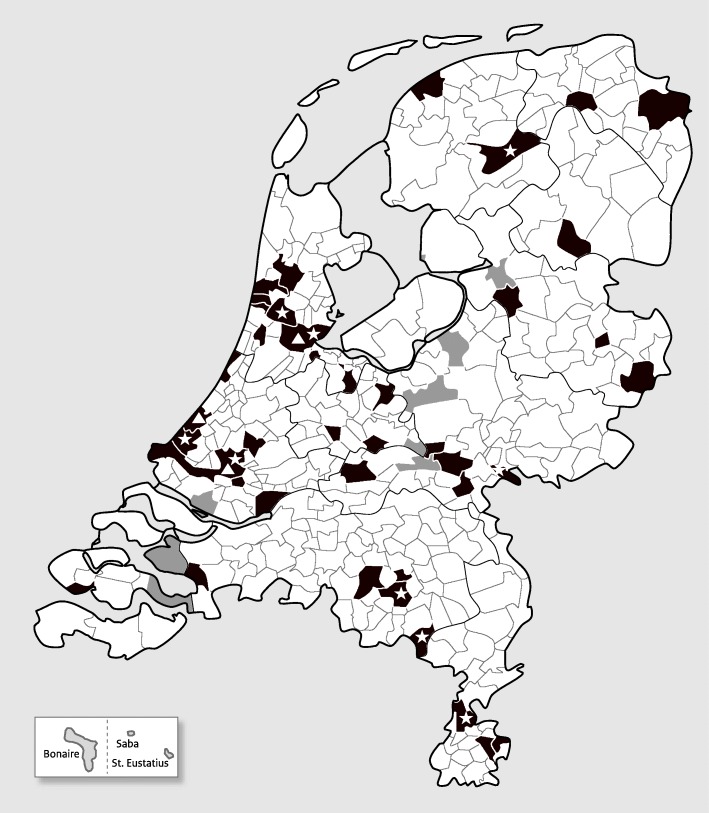
Overview of the selected municipalities. Municipalities depicted in black are included in the national sample and municipalities in grey are low vaccination coverage areas. * indicate a municipality with oversampling of non-Western migrants, ▲ indicate a municipality with oversampling of non-Western migrants and oversampling of people with migration background from Suriname, Aruba and the former Dutch Antilles. The Caribbean Netherlands sample, taken from the Dutch Caribbean islands Bonaire, St. Eustatius and Saba, are shown at the bottom left

Similar to the previous serum banks, a two-stage cluster sampling technique was used to draw a national sample (NS) in NL [1, 2]. Forty municipalities were sampled within five regions proportional to size (Fig. 1). Within each of these municipalities, an age-stratified sample was drawn from the population register. As life expectancy is increasing, the maximum age in this study was extended from 79 (in the previous surveys) to 89 years, resulting in age strata 0, 1–4, 5–9, …, up to 75–79, 80–89 years of age. A detailed description of the sample size calculations and total number of invitees per study sample can be found in Additional file 1: Table S1. In total, we aimed for 158 participants per municipality, resulting in 6320 participants. Therefore, initially, a sample of in principal 494 individuals per municipality was drawn in the first 11 municipalities, however during the study this was adjusted for the age strata 0–54 years because of lower response rate than expected, which resulted in a total of 818 persons invited in the next 13 municipalities. Finally, in the last 16 municipalities 193 extra men (in total 1011 persons) were invited in the age range of 20–54 year since women responded predominantly. In total 32,244 individuals were planned to receive an invitation in the Dutch national sample.

#### Oversampling of subpopulations

First, people with a migration background from non-Western countries living in NL (i.e., migrants) were oversampled using age strata 0–9, 10–34, 35–59, 60–89 years. Age-stratification was based on the NIP-ages, i.e., NIP-vaccines (except for human papillomavirus) are administered before the age of 10 years and those below the age of 59 were eligible for the routine NIP (introduced in 1957). A random sample of migrants from Turkey/Morocco, Suriname/Aruba/former Dutch Antilles, and other non-Western countries was drawn within nine municipalities of the NS (Additional file 1: Table S1). In total, 8259 migrant individuals were planned to receive an invitation in this sample. Note, migrants were also invited in the NS as we sampled at random regardless country of birth.

Second, an additional sample of 7328 individuals was drawn from persons with a migration background from Suriname, Aruba and the former Dutch Antilles (SAN), living in the largest municipalities in the NS sample, to be able to compare them with participants from CN (as described at the end of this section) (Additional file 1: Table S1).

Third, persons living in low vaccination coverage (LVC) areas in NL were oversampled. In these areas Orthodox Reformed individuals (ORI), who (partly) refuse vaccination based on religious grounds, live socio-geographically clustered. Eight areas were selected from which the vaccination coverage for diphtheria-tetanus-pertussis-polio (DTaP-IPV) and MMR was below 85% in the years between 2010 and 2014 (nationwide coverage > 90%) [16]. A sample was drawn in similar age strata as for migrants (0–9, 10–34, 35–59 and 60–89 years) (Additional file 1: Table S1). An extra municipality was added halfway the study to reach a sufficient number of participants living in LVC areas. In total, 6864 persons living in LVC areas were planned to be invited.

In summary, we planned to invite 54,695 persons in total for NL, however, due to incorrect data from the municipal health register, eventually the number of invited individuals was 54,170.

Last, for CN, a sample was drawn from the population registry of the Dutch overseas territories (PIVA-V, January 1, 2017). Of each island, an age-stratified sample with age strata 0–11, 12–17, 18–34, 35–59 and 60–89 years was drawn (Additional file 1: Table S1). All children aged < 18 years on St. Eustatius (*n* = 744) and Saba (*n* = 339) were approached to take part in the study in order to meet the power requirements. Students from Saba University School of Medicine were a priori excluded from the total population (*n* = 245) as they are non-permanent residents of Saba. In total, 8068 individuals were invited (Bonaire *n* = 4798; St. Eustatius *n* = 2135; and Saba *n* = 1135).

### Data collection

In NL, data collection took place from February 1, 2016 to October 16, 2017 and on CN from May 2, 2017 to June 19, 2017. Each person received an invitation letter by mail along with a brochure containing information on the study and an informed consent form. For the Dutch migrant populations, the invitation letter contained a reference to the translated invitation letter on the website and a supplementary instruction flyer was sent in English, Turkish, Arabian and French. On CN, all research material was available in the four most common languages on the islands: Papiamentu, English, Dutch and Spanish.

The invitees in NL were asked to fill in an online questionnaire and to visit a consultation hour in their municipality. A paper questionnaire was sent to people above 60 years of age. At the consultation hour, a shortened translated version of the questionnaire was available in English, Turkish, Arabian and French in case the invitee was not able to fill in the Dutch questionnaire. The invitees in CN received a paper questionnaire in their preferred language (Papiamentu, English, Dutch or Spanish) at the consultation hour and were guided by a trained interviewer in case of illiteracy or on request by the participant. The questionnaire of CN had some minor differences and was longer as compared to NL. Paper questionnaires and completed diaries were registered and digitized by trained data typists.

All invitees received a pre-made appointment for a consultation hour (to control the flow of visitors), however it was clarified in the letter that they were able to visit at any moment. Invitees in NL received a reminder by mail and were contacted via telephone by a call centre a few days prior to the visit. Invitees who did not show up were contacted by phone again. The call centre conducted a non-response questionnaire if a person was not willing to participate.

Several communication tools were applied to promote the study, such as twitter, radio- and television interviews and newspapers. Websites were operational for information and invitees could e-mail or call the research team with questions and/or consult an independent general practitioner if they preferred. Especially for CN, an extensive communication plan was composed in collaboration with all relevant stakeholders and was tailor-made for each island. At the start of the study a press conference and official kick-off was organised on each island.

At the consultation hours several body materials were collected. In NL, the collection consisted of saliva and venous blood samples. For babies a heel prick was offered and for young children or people who were dreading blood donation a finger prick was available. A small subset of the participants (*n* = 338) was asked to donate an extra blood sample for cellular immunity analyses and a subset (*n* = 1939) was asked to fill in a dairy about contact patterns. Moreover, participants could optionally donate additional materials for which they received an extra incentive. These materials included oro- and nasopharyngeal swabs (for children below the age of 8 years solely one swab was required, preferably a nasopharyngeal swab), and a faecal sample including an additional questionnaire. These participants were also asked for their consent regarding retrieval of their complete medication history – through collaboration with Dutch pharmacies (the UPPER-network) and the Foundation for Pharmaceutical Statistics (‘Stichting Farmaceutische Kerngetallen’). In CN, anthropometric measurements height, weight and blood pressure (the latter from 4 years of age) were taken via calibrated instruments (SECA 214, SECA 877, and Omron M3, respectively) and standardized methods. If height and weight were not able to be assessed this was acquired from the latest measurement (growth booklet) at the Public Health Services or estimated by the participant. Thereafter, blood samples – via a finger- or heel prick using the dried blood spot (DBS) method – and oro- and nasopharyngeal swabs were collected. Participants were asked to collect a faecal sample at home and were offered a gift voucher after returning their sample. Permission was asked for retrieval of their medication history of the preceding year from the local health insurance office (‘Zorgverzekeringskantoor’). All participants in NL and CN were asked for their consent regarding participation in a possible follow-up research (nested cohort).

The vaccination history of the participants was either checked by copying the vaccination certificates brought by the individuals to the consultation hour or retrieved via Praeventis: the Dutch electronic (web-based) vaccination register of the NIP for birth cohorts from 1990 on. For participants born before 1990 in NL, vaccination histories were retrieved via former local authority for registration of vaccinations. In CN, the vaccination statuses were additionally obtained via Public Health Services, consultation offices and hospitals.

Invitees older than 6 years of age who were not able to visit the consultation hour or did not show up at the clinic in NL, yet filled in (part) of the (online) questionnaire, were sent a kit to self-administer a finger prick sample at home. Likewise, people who were willing to participate but were not able to come to the clinic were sent a finger prick-kit in NL or were visited at home in CN.

Information on environmental exposure at the address level (e.g., different parameters for air pollution (PM10, PM2.5, livestock-associated air pollution) and green space) were retrieved from national databases in NL [17–19]. The linkage to these environmental parameters and the medication histories enables investigation of effects on the microbiome and antibiotic resistance at varies sites. In combination with results from sera and oro- and nasopharyngeal swabs the association of vaccination responses and carriage of pathogens, microbiome, environmental and lifestyle factors can be investigated.

The study proposal was approved by the Medical Ethics Committee Noord-Holland (METC number: M015–022) and written informed consent was obtained from all adult participants, and parents or legal guardians of minors included in the study.

### Processing and storage of body materials

In NL, collected materials were transported to the RIVM’s laboratory at the end of each consultation day. In adults, blood was drawn in two 8.5 ml vacutainer tubes (Becton and Dickenson SST II) and one 2 ml EDTA blood tube (total of max. 19 ml), and in children until the age of five years a maximum of 10 ml of blood was drawn. Heel- or finger pricks were collected in 300 μl cups. In the subset of participants who also donated blood for cellular immunity analyses, additional blood was drawn in two 9 ml heparin tubes. The blood samples were stored in a cold room (4 °C) overnight at the RIVM. The next day, the 2 ml EDTA blood tube was registered and stored in the freezer (− 20 °C). The blood collected in vacutainers was centrifuged and divided into portions up to 4.5 ml serum. One tube of serum was stored at − 20 °C (for further aliquoting) and the remaining serum, if available, was stored at − 80 °C. Heel- and finger prick blood was centrifuged, aliquoted, and stored in − 20 °C. The blood collected in heparin tubes was used for whole blood phenotyping by FACS analyses, the plasma was stored in aliquots in − 20 °C and PBMCs were isolated and stored in vials at − 135 °C. Saliva samples were collected using a sponge that was swabbed through the mouth for one minute. Saliva was immediately harvested from the swab by squeezing fluids from the sponge and dividing the sample into a cryovial® tube and a tube containing a glycerol solution (50% glycerol in DEPC water, for culture). These two tubes were immediately frozen on dry-ice at the consultation hour and stored the next day at − 80 °C. The remaining saliva was collected into a 2 ml spray dried EDTA tube and stored at room temperature. The next morning the samples were centrifuged, aliquoted and stored at − 80 °C.

Oro- and nasopharyngeal swabs were taken and stored in liquid Amies medium. The swabs were immediately frozen at the consultation hour on dry-ice and transported at the end of the consultation day to the RIVM, where they were stored the next day at − 80 °C. For the collection of faecal material, subjects were requested to donate a small amount of faeces in three separate containers at home, of which one contained 15% glycerol-physiological salt solution. The samples were then packed in a plastic bag directly after collection and kept in the freezer at home until they were delivered by the subject in cold packs to the mobile study team. Detailed instructions and all materials needed were supplied at the first visit at the consultation hour. Faecal samples were kept frozen on dry-ice during transport to the RIVM and stored at − 80 °C the next day.

All samples collected in CN were stored at, preferably air-conditioned, room temperature at the consultation hour. Blood samples were collected via a finger -or heel prick using the DBS method via air-dried filter paper (Whatman® 903 protein saver cards), removing barriers related to sample collection and transportation. These were dried for a minimum of two hours before storage in plastic bags with silica pads. Oro- and nasopharyngeal swabs were collected and stored in a 1 ml MMB tube (DNA Genotek Inc., Ottawa, Canada) and faecal samples in a 1 ml OMNIgene®-gut (OMR-200) (DNA Genotek Inc.) tube, both containing stabilizing liquid for the microbiome. Directly after the fieldwork, samples were air shipped to the laboratory of the RIVM where the material was stored instantly at − 80 °C pending analyses.

## Results

### The Netherlands

From the 54,170 people invited, 195 (0.4%) persons were excluded from the sample for not having received the invitation (due to rehousing, no delivery or other reasons, *n* = 186) or due to mental disability (*n* = 9). This resulted in 53,975 (99.6%) invitees: 39,898 within the NS, including 8184 oversampled non-Western migrants, 6825 from LVC areas and 7252 oversampled people with SAN background.

In total, 7600 (14.1%) sera and questionnaires were collected in NL: 5745 (response rate 14.4%, range 4.9–21.9 per municipality) in the NS including the extra sample of migrants (*n* = 601, response 7.3%), 1354 in the LVC sample (response rate 19.8%, range 15.0–26.3 per municipality) and 501 (6.9%) persons in the SAN sample. A detailed description of response per study sample, stratified by age groups can be found in Additional file 1: Table S1. An overview of collected materials per sample is shown in Table 2. Moreover, 5105 (82.1%) participants with any material in the NS gave consent to be approached for a follow-up study if applicable. Of all non-responders, 15,141 (77.0%) answered the question concerning their reason not to participate: 36.0% indicated that they did not have time to participate, 12.0% was dreading blood donation and 45.0% gave a reason other than the above mentioned answer categories and 7.0% did not answer this question.

**Table 2:** Overview of number of participants and collected materials in the study, by sample (n (%))

	NS & oversampling of migrants	LVC sample	SAN sample	CN sample
Sample invited	40,065	6830	7275	8068
Net sample size^a^	39,898 (99.6%)	6825 (99.9%)	7252 (99.7%)	7768 (96.3%)
Information from population register only	19,638 (49.0%)	2436 (35.7%)	4040 (55.5%)	NA
Non-response questionnaire	14,043 (35.2%)	2964 (43.4%)	2652 (36.6%)	NA
Participant with any material	6217 (15.6%)	1425 (20.9%)	560 (7.7%)	1900 (24.5%)
Full participant (all materials)	2682 (6.7%)	674 (9.9%)	280 (3.7%)	1515 (19.5%)
Participant with both blood sample and questionnaire	5745 (14.4%)	1354 (19.8%)	501 (6.9%)	1815 (23.4%)
Materials^b^				
Blood^c^ - Venous blood sample - Finger/heel prick - Dried blood spot sample	5762 (92.7%) 4977 (86.4%) 581 (10.1%) 208 (3.6%)	1358 (95.3%) 1174 (86.5%) 162 (11.9%) 23 (1.7%)	503 (89.8%) 454 (90.3%) 25 (5.0%) 26 (5.2%)	1829 (96.3%) NA NA 1829 (100%)
Questionnaire	6200 (99.7%)	1421 (99.7%)	558 (99.6%)	1885 (99.2%)
Saliva sample	5544 (89.2%)	1319 (92.6%)	477 (85.2%)	NA
Nasopharyngeal swab	3849 (61.9%)	939 (65.9%)	369 (65.9%)	1752 (92.2%)
Oropharyngeal swab	3319 (53.4%)	791 (55.5%)	326 (58.2%)	1502 (79.1%)
Faeces	2765 (44.5%)	704 (49.4%)	285 (50.9%)	1547 (81.4%)
Additional questionnaire	2775 (44.6%)	702 (49.3%)	284 (50.7%)	NA
Vaccination status^d^	3819 (71.1%)	970 (76.3%)	263 (57.9%)	974 (51.3%)
Diary contact patterns^e^	1310 (72.7%)	NA	67 (48.9%)	NA
Consent to approach for follow-up^d^	5105 (82.1%)	1171 (82.2%)	436 (77.9%)	1762 (92.7%)

^a^Reasons for exclusion included mentally disabled, died, rehousing or other reasons why mail could not be delivered

^b^Percentages were calculated with participants with any material as denominator

^c^Four, one and two person(s) with both finger prick and venous blood in NS, LVC sample and SAN sample, respectively

^d^Percentages were calculated for participants with any material and eligible for the NIP programme (<=65 years), 5374, 1271, and 454 for NS, LVC, and SAN sample, respectively. For the CN sample, percentage was calculated for all participants with any material

^e^Percentages were calculated with number of diaries handed out as denominator, 1802 and 137 in NS and SAN sample, respectively

Abbreviations: *CN* sample, Caribbean Netherlands: sample from the Dutch Caribbean islands Bonaire, St. Eustatius and Saba; *LVC* sample, Low vaccination coverage sample; *NA*, Not applicable; *NS*sample, National sample; *SAN* sample, Sample from persons with a migration background from Suriname, Aruba and the former Dutch Antilles

Additional file 2: Table S2 shows the frequencies on a set of socio-demographic characteristics for responders (participants with any material) versus non-responders for the NS sample (including the oversampling of non-Western migrants) and the CN sample. Among the responders there are relatively more persons aged between 10 and 19 years and between 40 and 79 years, more women, more indigenous Dutch people, and less people living in areas with the highest degree of urbanisation compared to non-responders.

**Fig. 2:**
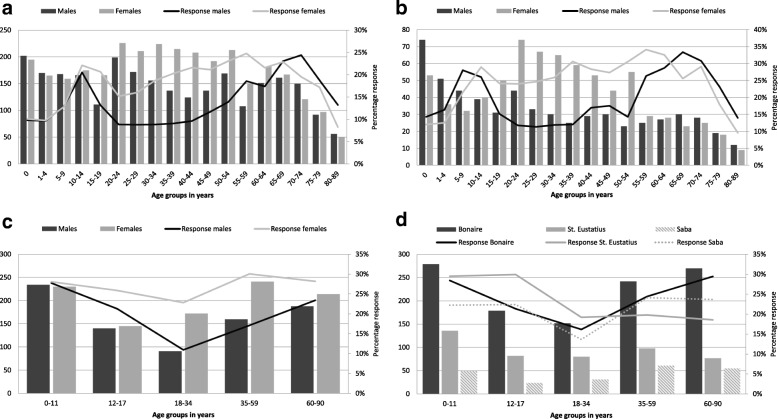
Overview of participants with both a blood sample and a questionnaire and corresponding response rates (n(%)). **a:** Overview of number of participants of the national sample and oversampling of non-Western migrants of the PIENTER-3 study, stratified by sex and age class. **b:** Overview of number of participants of the low vaccination coverage sample of the PIENTER-3 study, stratified by sex and age class. **c:** Overview of number of participants of the Caribbean Netherlands sample, stratified by sex and age class. **d:** Overview of number of participants of the Caribbean Netherlands sample, stratified by island and age class

Overall, more females (54.7%) responded than males (45.3%) (Table 3), however in the youngest and highest age classes more males participated. In both the NS (Fig. 2a) and LVC sample (Fig. 2b) the highest response rate was seen in women aged 55–59 years. The high number of invited 20–54-year-olds resulted in a high inclusion of females from this age class.

**Table 3:** Overview of socio-demographic characteristics for participants with both a blood sample and questionnaire, by sample (*n* (%))

	NS & oversampling of migrants	LVC sample	CN sample
	5745 (14.4%)	1354 (19.8%)	1815 (23.4%)
Sex	5745 (100.0%)	1354 (100.0%)	1815 (100.0%)
Male	2629 (45.8%)	594 (43.9%)	814 (44.8%)
Female	3116 (54.2%)	760 (56.1%)	1001 (55.2%)
Ethnicity	5744 (99.9%)	1354(100.0%)	1804 (99.4%)
Indigenous Dutch	4490 (78.1%)	1299 (96.0%)	146 (8.1%)
Morocco and Turkey	142 (2.5%)	3 (0.2%)	1 (0.1%)
Suriname, Aruba and former Dutch Antilles	285 (5.0%)	4 (0.3%)	1301 (72.1%)
Other non-Western countries	445 (7.7%)	15 (1.1%)	280 (15.5%)^a^
Other Western countries	382 (6.7%)	33 (2.4%)	76 (4.2%)
Religion	5330 (92.8%)	1200 (88.6%)	1784 (98.3%)
Protestant	841 (15.8%)	843 (70.3%)	44 (2.5%)
- Orthodox Reformed	79 (9.4%)	299 (35.5%)	NA
Roman Catholic	1279 (24.0%)	29 (2.4%)	892 (50.0%)
Other religion	598 (11.2%)	44 (3.7%)	625 (35.0%)
No religion	2612 (49.0%)	284 (23.7%)	223 (12.5%)
Educational level^b^	5407 (94.1%)	1280 (94.5%)	1574 (86.7%)
High educational level	1465 (27.1%)	443 (34.6%)	319 (20.3%)
Middle educational level	1855 (34.3%)	540 (42.2%)	401 (25.5%)
Low educational level	2087 (38.6%)	297 (23.2%)	854 (54.2%)
Urbanisation degree^b^	5745 (100.0%)	1354 (100.0%)	NA
1 (Highly urbanised)	1246 (21.7%)	–	NA
2 (Urbanised)	1873 (32.6%)	–	NA
3 (Moderate urbanised)	1090 (19.0%)	159 (11.7%)	NA
4 (Little urbanised)	1041 (18.1%)	753 (55.6%)	NA
5 (Countryside)	495 (8.6%)	442 (32.6%)	NA

^a^In the CN sample *n* = 260 of 280 (93%) participants from ethnic group ‘other non-Western countries’ had a Latin American background. Total proportion of Latin Americans in the CN sample is 14.4%

^b^Definitions according to Statistics Netherlands (CBS). Urbanisation degree is based on the environmental address density municipalities are divided into five classes of urbanity. The environmental address density is the average value of the address density of a municipality. The address density is based on an area with a radius of 1 km around an address

Abbreviations: *CN* sample, Caribbean Netherlands: sample from the Dutch Caribbean islands Bonaire, St. Eustatius and Saba; *LVC* sample, Low vaccination coverage sample; *NA*, Not applicable; *NS* sample, National sample

Nearly half of the people (49.0%) in the NS were not religious, 24.0% considered themselves Roman Catholic, 15.8% Protestant, and 11.2% reported to have another religion, such as Islamic, Jewish, Buddhism or Hinduism (Table 3). Most participants in the NS were low educated (38.6%), followed by middle (34.3%) and high (27.1%). Moreover, the highest response was seen in indigenous Dutch and the lowest in individuals with a Moroccan or Turkish background, in which the anticipated 70 persons per age strata were not reached. Regarding the other migrant groups, over 70 participants per age group were included, except for the 0–34-year-olds with a migration background from Suriname/Aruba/former Dutch Antilles (0–9 year: *n* = 44; 10–34 year: *n* = 62). Nevertheless, as the total number of participants with a SAN background (including *n* = 501 in the SAN sample) was 786, sufficient participants were included in each age group.

In the LVC sample, the largest amount of people considered themselves Protestant (*n* = 844, 70.3%) and among them 299 (35.4%) were ORI (Table 3). Together with those included in the NS, a total of 378 ORIs were included, in which all age strata included over 70 persons, except for 60–89-years-old (*n* = 49). Mainly middle- and high-educated people were included in the LVC sample; 23% of the LVC sample was low educated.

### Caribbean Netherlands

Of 8068 invitees, 300 (3.7%) were excluded from the sample for not having received the invitation (due to rehousing or because they were unknown on the address (*n* = 196), or because of delivery issues (*n* = 84)), mental disability (*n* = 17) or death (*n* = 3). Table 2 shows the participants and collected materials in CN. In total, 1815 persons (23.4%) completed the questionnaire and donated a blood sample (Bonaire: 1122 (24.0%); St. Eustatius: 473 (22.9%); Saba: 220 (21.2%)), and 1762 (92.7%) participants with any material gave consent to be approached for a follow-up study. A detailed description of response per island, stratified by age groups, can be found in Additional file 1: Table S1. Among the responders there are relatively more women, more persons aged 0–11 years old and fewer people aged 18–34 years old compared to the non-responders (See Additional file 2: Table S2).

In total, more females (55.2%) were included than males (44.8%) (Table 3). Females responded better than males on each island, with 27.1% vs. 20.0% on average in CN, respectively (Fig. 2c and Fig. 2d). More specifically, the highest response was seen in females aged 35–59 years (30.0%) and the lowest in males aged 18–34 years (11.0%). As for country of birth, invitees born in Aruba and the former Dutch Antilles (24.4%) as well as in NL (28.3%) responded significantly better compared to participants born in another country (18.9%), especially on St. Eustatius and Saba (data not shown). Half of the people included considered themselves as Roman Catholic, a small portion as Protestant (2.5%), 35% as other than the previous two, such as Methodist or Adventist, and 12.5% indicated not to be religious. Moreover, more than half of the participants (54.2%) indicated to be lower educated, followed by middle (25.5%) and high (20.3%).

## Discussion

A third national biobank among the general population of NL has been generated and will be an important tool to evaluate infectious disease control in NL and contribute to public health policy. The seroprevalence data will provide insight into the effectiveness of the Dutch NIP and direction for improvement. These results will also be of value for future outbreak management. In addition, data collection has been extended to CN for the first time, which resulted in an extensive amount of information that will enable us to support future public health policy on these Caribbean islands, e.g., regarding tropical pathogens. Moreover, besides serum collection, a large number of additional materials have been collected, which allows us to look into relevant emerging topics, such as antibiotic resistance and the microbiome. Hence, this biobank offers unique opportunities to investigate infectious diseases in a much broader sense.

A high number of persons participated in this study, which enables us to perform most seroepidemiological (sub)analyses with sufficient power as calculated beforehand. Response rate (blood and questionnaire) in the NS sample was 14.4% and in CN 23.4% and, in line with other international studies [20–22], females and elderly as well as natives were highest responders among the invitees. Overall, the NS sample (including the oversampling of non-Western migrants) is rather comparable to the Dutch population, especially regarding urbanisation degree and religion [23, 24]. Religion is an important variable with regard to seroprevalence as vaccination coverage among several denominations in NL is much lower compared to the rest of NL [16]. Females as well as indigenous Dutch, people from SAN and other non-Western countries – other than Morocco and Turkey – were slightly overrepresented (e.g., 54.2% females and 78.1% indigenous Dutch in our sample vs. 50.4% and 73.2%, respectively, in the Dutch population) [25]. Overrepresentation of SAN and non-Western migrants was due to the oversampling. Further, participants had a lower educational level as compared to the general Dutch population (31.4% is low-educated in the Dutch population vs. 38.6% in our sample) [26]. Generally, for each age strata in the NS sample, sufficient participants were included, although overall we reached marginally lower numbers per age strata than in the 2006/2007 study, except for 0-year-olds. For the LVC sample, sufficient ORI-participants were included in each age strata, except for the oldest age group (60–89 years).

The CN sample in this study is generally a good reflection of the total population on the islands concerning religion and educational level [27, 28]. People born in non-Western countries (which consists of 93% Latin Americans in our CN sample) and males are slightly less represented in our study population though (namely, 14.4% Latin Americans and 44.8% males in our sample vs. 19.3% and 51.5%, respectively, in the CN population) [29].

Socio-demographic dissimilarities compared with the general populations of NL and CN, due to selectivity in response and the sample design, will be taken into account by weighting the participants on a set of variables (age, sex, ethnicity and degree of urbanisation). An in-depth non-response analysis for NL will be carried-out and published in the near future. Moreover, having applied an identical robust design for the third time ensures maximum comparison with previous studies and opens opportunities for changes over time and modelling analyses. A less costly and less extensive design of sample collection, for instance via residual sera, is more prone to selective response and lacks the opportunity to collect additional materials as well as data on various characteristics of participants to perform risk factor analyses.

The response rate in the NS sample (14.4%) was lower as compared to the two previous serosurveys performed in NL (50% in 1995/1996 and 32% in 2006/2007). Nonetheless, low(er) response rates have also been reported in other recently conducted large population-based studies in NL (e.g., ‘NL de Maat genomen’, phase 1: < 20% [30] and ‘Lifelines’: 24.5% [31]) as well as abroad, for instance in the United Kingdom (‘UK biobank’ 5.5% [32]). Likewise, participation in health examination studies in other European countries (e.g., ‘FINRISK’, Finland, ‘HSE’, England, ‘DEGS’, Germany, etc.) all show a decrease in responses over the past decades [21] and this declining trend is also observed in a large serosurveillance study in America (‘NHANES’) [20]. Although the underlying reasons for this worldwide decreasing trend in study participation is not exactly known and may differ per study and country, our non-response questionnaire indicated that most persons did not have time to come to the clinic or were dreading blood collection. It has been previously reported that participation in a population-based survey including collection of blood, especially in children, is likely to be low [33, 34]. Although we tried to organize several consultation hours at centralized locations, travel time might have also been a conflicting factor for some people, especially in larger municipalities where response rates were lowest. Other sampling options, such as self-sampling finger pricks or house visits, could therefore be considered in the future to overcome some of the hurdles of reluctance to participate. This implies, however, that laboratory techniques should be suitable for analyses in low volumes of blood or other sources of material, like DBS or saliva. Last, as participants often do not receive any personal results when participating in health studies, we suspect that, in nowadays more individualized societies, there might be less willingness to contribute without direct personal benefit; hence, future studies should consider such incentives if feasible.

The response rate in CN (23.4%) was higher compared to NL. Possible explanations might be a high awareness of the study as a relatively high proportion of island residents were invited to participate, the extensive local media attention, and the presumably less individualistic island culture. Previous studies on the islands reported higher participation rates (e.g., ‘Omnibusenquête’ (2013) [35]: 40–62%; ‘Kon Salu ta…’ (‘How healthy is…’) (2002): 80–86%) [36–38], however a completely different approach was used in these studies: house visits with multiple contact efforts, the design (solely questionnaire), and longer duration of the study, i.e., our efficient time planning limited adjustment during the study in order to increase response. Further, the population registry was not fully up-to-date and the delivery of the invitations by mail was challenging for the local postal department, which both could have negatively affected our net response. This reaffirms that logistical matters are unavoidable for these small islands and thus future studies should consider building in large(r) time margins. Moreover, population-based studies including sample collection are unfamiliar among the CN population. In order to increase awareness and response, an extensive communication plan was made tailored per island using all communication tools available. We experienced that personal appeal, repetition and promotion via key figures was most beneficial in term of response. Nevertheless, initially we might have missed some participants due to taboos towards collection of faecal material. In line with our study in NL, future studies in CN could consider introducing additional collection with ditto incentive regarding such samples to increase overall response.

## Conclusions

In conclusion, this third Dutch biobank offers unique future research possibilities. Over 9000 blood samples and questionnaires as well as additional materials, such as saliva, oro- and nasopharyngeal swabs and faeces, have been collected. This offers the opportunity to perform thorough seroprevalence studies assessing immunity against and risk factors for (candidate) NIP-targeted diseases, taking into account different infection dynamics in the Caribbean region. Furthermore, for the first time, these data will inform us on the occurrence of and risk factors for tropical pathogens in the Caribbean region, such as zika, dengue, chikungunya, West Nile virus and yellow fever. Moreover, we will be able to connect the serological data to results from additional materials collected (via molecular typing and bacterial cultures) and environmental- and pharmacological data. Hence, we are able to gain relevant new insights into the emerging fields of the microbiome, antibiotic resistance and carriage of pathogens in relation to vaccination responses, allergies, environmental- and lifestyle factors [39]. Besides the extensive data collection, the vast majority of participants gave consent for participation in a potential follow-up study enabling a nested-case cohort study in the future. Summarized, this large biobank forms a great base for research in the field of infectious disease epidemiology and its changing dynamics and consequently, this knowledge can guide future public health policy in NL and CN.

## Additional files

Additional file 1: **Table S1.** Supplementary information regarding sample size calculations, number of invitees and response of the PIENTER-3 study, stratified by study sample. (DOCX 46 kb)

Additional file 2: **Table S2.** Supplementary information regarding response of the PIENTER-3 study, stratified by study sample. (DOCX 23 kb)

## Abbreviations

CN: Caribbean Netherlands
DBS: Dried blood spot
DTaP-IPV: Diphtheria-tetanus-pertussis-polio
HSCN: Health Study Caribbean Netherlands
LVC: Low vaccination coverage
MMR: Measles-mumps-rubella
NIP: National Immunization Programme
NL: The Netherlands
NS: National sample
ORI: Orthodox Reformed Individuals
PIENTER: Seroepidemiological study in the Netherlands to monitor the effect of the National Immunization Programme (in Dutch: ‘Peiling Immunisatie Effect Nederland Ter Evaluatie van het Rijksvaccinatieprogramma’)
PIVA-V: Population registry of the Dutch overseas territories
RIVM: National Institute for Health and the Environment (in Dutch: ‘Rijksinstituut voor Volksgezondheid en Milieu’)
SAN: Suriname, Aruba and the former Dutch Antilles
