# Supplementary material for: Third national biobank for population-based seroprevalence studies in the Netherlands, including the Caribbean Netherlands
Source: BMC Infect Dis. 2019 May 28;19:470. doi: 10.1186/s12879-019-4019-y (PMC6537387; doi:10.1186/s12879-019-4019-y)
Supplement: Supplementary file 1 — Table S1. Supplementary information regarding sample size calculations, number of invitees and response of the PIENTER-3 study, stratified by study sample. (DOCX 46 kb) [file 12879_2019_4019_MOESM1_ESM.docx]

Additional file 1: Table S1

Supplementary information regarding sample size calculations, number of invitees and response of the PIENTER-3 study, stratified by study sample.

| **Sample** | **Age-specific pre-valence** | **Precision**  **(one-way test)** | **Alpha error** | **Anticipated response rate** | **Number of invitees (anticipated number of participants)** | **Number of participants with a questionnaire and blood sample (response %)*** | **Remarks** |
| --- | --- | --- | --- | --- | --- | --- | --- |
| National sample **(NS)** | 50% | 2.5% overall seroprevalence and 10-15% age-specific seroprevalence | 5% | The starting point was a similar number of invitees per municipality as in the former study and response rates of NS sample in previous study (PIENTER-2)^1^ thereby oversampling 0-4-year-olds and 20-39-year-olds because of lower response rates | 0y: 4059 (400)  1-4y: 2557 (400)  5-9y: 1410 (480)  10-14y: 1308 (480)  15-19y: 1348 (360)  20-24y: 3441 (360)  25-29y: 2883 (360)  30-34y: 2528 (320)  35-39y: 2129 (360)  40-44y: 1841 (320)  45-49y: 1693 (360)  50-54y: 1767 (360)  55-59y: 880 (360)  60-64y: 880 (400)  65-69y: 880 (400)  70-74y: 880 (320)  75-79y: 880 (280)  80-89y: 880 (280)  Total:32,244 (6320) | 393 (10%)  288 (11%)  240 (17%)  314 (24%)  256 (19%)  408 (12%)  362 (13%)  350 (14%)  321 (15%)  303 (17%)  294 (18%)  348 (20%)  221 (26%)  261 (30%)  271 (31%)  239 (28%)  178 (21%)  97 (11%)  5,144 (16%) | In the first 11 municipalities a sample of in principal 494 individuals per municipality was drawn, thereby oversampling 0-year-olds and 5-19-year-olds to calculate seroprevalence for each four months in the first year of life and per year until the age of 19 years. However, during the study this was adjusted for the age strata 0-54 years because of lower response rate than expected, which resulted in a total of in principal 818 persons invited in the next 13 municipalities based on the actual inclusion rates. Finally, in the last 16 municipalities 193 extra men (a total of in principal 1011) were invited in the age range of 20-54 year since women responded predominantly. |
| Oversampling non-Western migrants **(migrants)** | 75% | 10% age-specific seroprevalence | 5% | Response rates of migrant sample in previous study (PIENTER-2)^1^ | 0-9y: 2167 (199)  10-34y: 1815 (167)  35-59y: 2017 (187)  60-89y: 2260 (207)  Total: 8,259 (760) | 138 (6.6%)  116 (6.2%)  166 (8.1%)  181 (8.4%)  601 (7.3%) | The number of invited migrants per municipality was in line with the distribution of the migrant groups per urbanisation degree in the Dutch population. We aimed for 70 participants per migrant group and age group. Note, that for the calculation of the number of extra non-Western migrants to invite in this sample we took into account the number of expected non-Western migrant participants in the NS sample based on response rates per migrant group and age group of the previous study (PIENTER-2). |
| Oversampling people in low vaccination coverage areas **(LVC)** | 75% | 10% age-specific seroprevalence | 5% | Response rates of LVC sample in previous study (PIENTER-2)^1^ | 0-9y: 1879 (70 ORIs)  10-34y: 2460 (70 ORIs)  35-59y: 1,625 (70 ORIs)  60-89y: 900 (70 ORIs)  Total: 6,864 (280 ORIs) | 84 ORIs (4.5%)  117 ORIs (4.8%)  65 ORIs (4.0%)  33 ORIs (3.7%)  299 ORIs (4.4%) | An oversampling of 0-year-olds took place, with the aim to include 50 Orthodox Reformed infants in total. For each of the four age strata we aimed to include 70 Orthodox Reformed Individuals (ORIs). An extra municipality was added halfway the study to reach a sufficient number of participants living in LVC areas. In the last municipality, the number of invited individuals was increased in the 20-54 year-old men. |
| Oversampling people with migration background from Suriname, Aruba and the former Dutch Antilles **(SAN)** | 50% | 10% age-specific seroprevalence | 5% | 7%, based on response rate SAN people first 10 municipalities NS | 0-9y: 1,833 (95)  10-34y: 1,833 (95)  35-59y: 1,832 (94)  60-89y: 1,830 (94)  Total: 7,328 (378) | 96 (5.3%)  79 (4.4%)  137 (7.6%)  189 (10.4%)  501 (6,9%) | The SAN group will be part of a specific serosurvey analysis. |
| Sample of Caribbean Netherlands **(CN)** |  |  |  |  | Total: 7768 (2442) | Total: 1815 (23%) |  |
| Bonaire | 50% | 5.5% age-specific seroprevalence | 5% | 30%, based on response rate NS sample in previous study (PIENTER-2)^1^ | 0-11y: 982 (284)  12-17y: 839 (258)  18-34y: 940 (296)  35-59y: 990 (305)  60-89y: 916 (289)  Total: 4,667 (1432) | 0-11y: 279 (28%)  12-17y: 179 (21%)  18-34y: 152 (16%)  35-59y: 242 (24%)  60-89y: 270 (29%)  Total: 1,122 (24%) | Samples were drawn using PIVA-V of January 1^st^, 2017. An additional sample of Bonaire was drawn using PIVA-V of April 1, 2017 to include all registered new-borns from January 1 till March 31, 2017 (n=42). |
| St. Eustatius | 50% | 7.5% age-specific seroprevalence | 5% | 30%, based on response rate NS sample in previous study (PIENTER-2)^1^ | 0-11y: 461 (126)  12-17y: 274 (107)  18-34y: 417 (136)  35-59y: 495 (151)  60-89y: 415 (132)  Total: 2,062 (652) | 0-11y: 136 (30%)  12-17y: 82 (30%)  18-34y: 80 (19%)  35-59y: 98 (20%)  60-89y: 77 (19%)  Total: 473 (23%) |  |
| Saba | 50% | 10% age-specific seroprevalence | 5% | 30%, based on response rate NS sample in previous study (PIENTER-2)^1^ | 0-11y: 220 (69)  12-17y: 107 (51)  18-34y: 227 (78)  35-59y: 253 (84)  60-89y: 232 (76)  Total: 1,039 (358) | 0-11y: 49 (22%)  12-17y: 24 (22%)  18-34y: 31 (14%)  35-59y: 61 (21%)  60-89y: 55 (24%)  Total: 220 (21%) |  |
| ^1^van der Klis FR, Mollema L, Berbers GA, de Melker HE, Coutinho RA. Second national serum bank for population-based seroprevalence studies in the Netherlands. The Netherlands journal of medicine. 2009;67(7):301-8.  *****For the response (%) percentages were calculated based on net response, i.e. excluding non-eligible invitees. | | | | | | | |
