# Supplementary material for: Third national biobank for population-based seroprevalence studies in the Netherlands, including the Caribbean Netherlands
Source: BMC Infect Dis. 2019 May 28;19:470. doi: 10.1186/s12879-019-4019-y (PMC6537387; doi:10.1186/s12879-019-4019-y)
Supplement: Supplementary file 2 — Table S2. Supplementary information regarding response of the PIENTER-3 study, stratified by study sample. (DOCX 23 kb) [file 12879_2019_4019_MOESM2_ESM.docx]

Additional file 2: Table S2

Supplementary information regarding response of the PIENTER-3 study, stratified by study sample.

**Responders versus non-responders in national sample, including the oversampling of non-Western migrants.**

|  | **Number of responders with any material (%)** | **Number of non-responders (%)** |
| --- | --- | --- |
| **Age groups** | 6,217 (100%) | 33,681 (100%) |
| 0y | 451 (7.3%) | 3,548 (10.5%) |
| 1-4y | 382 (6.1%) | 3,068 (9.1%) |
| 5-9y | 354 (5.7%) | 2,182 (6.5%) |
| 10-14y | 367 (5.9%) | 1,233 (3.7%) |
| 15-19y | 313 (5.0%) | 1,331 (4.0%) |
| 20-24y | 469 (7.5%) | 3,269 (9.7%) |
| 25-29y | 429 (6.9%) | 2,843 (8.4%) |
| 30-34y | 422 (6.8%) | 2,541 (7.5%) |
| 35-39y | 380 (6.1%) | 2,188 (6.5%) |
| 40-44y | 361 (5.8%) | 1,894 (5.6%) |
| 45-49y | 351 (5.7%) | 1,735 (5.2%) |
| 50-54y | 405 (6.5%) | 1,734 (5.2%) |
| 55-59y | 273 (4.4%) | 915 (2.7%) |
| 60-64y | 342 (5.5%) | 1,372 (4.1%) |
| 65-69y | 334 (5.4%) | 1,096 (3.3%) |
| 70-74y | 277 (4.5%) | 957 (2.8%) |
| 75-79y | 193 (3.1%) | 862 (2.6%) |
| 80-89y | 114 (1.8%) | 913 (2.7%) |
| **Sex** | 6,217 (100%) | 33,681 (100%) |
| Males | 2,859 (46.0%) | 18,756 (55.7%) |
| Females | 3,358 (54.0%) | 14,925 (44.3%) |
| **Country of birth** | 6,217 (100%) | 33,681 (100%) |
| The Netherlands | 4,827 (77.2%) | 19,586 (58.2%) |
| Morocco + Turkey | 162 (2.6%) | 3,901 (11.6%) |
| Suriname+Dutch Antilles+Aruba | 323 (5.2%) | 3,334 (9.9%) |
| Other non-Western countries | 489 (7.9%) | 3,954 (11.7%) |
| Other Western countries  Unknown | 415 (6.7%)  1 (0.1%) | 2,884 (8.6%)  22 (0.1%) |
| **Degree of urbanisation** | 6,217 (100%) | 33,681 (100%) |
| 1 Highly urbanised | 1,369 (22.0%) | 11,475 (34.1%) |
| 2 Urbanised | 2,027 (32.6%) | 10,142 (30.1%) |
| 3 Moderate urbanised | 1,179 (19.0%) | 5,523 (16.4%) |
| 4 Little urbanised | 1,116 (18.0%) | 4,156 (12.3%) |
| 5 Countryside | 526 (8.5%) | 2,385 (7.1%) |

**Responders versus non-responders in sample of Caribbean Netherlands (CN).**

|  |  | **Number of responders with questionnaire and blood (%)** | **Number of non-responders (%)** |
| --- | --- | --- | --- |
| **Island** | | 1,815 (100%) | 5,953 (100%) |
|  | Bonaire | 1,122 (61.8%) | 3,545 (59.5%) |
|  | St. Eustatius | 473 (26.1%) | 1,589 (26.7%) |
|  | Saba | 220 (12.1%) | 819 (13.8%) |
| **Sex** | | 1,815 (100%) | 5,953 (100%) |
|  | Males | 813 (44.8%) | 3,260 (54.8%) |
|  | Females | 1,002 (55.2%) | 2,693 (45.2%) |
| **Age groups** | | 1,815 (100%) | 5,953 (100%) |
|  | 0-11y | 464 (25.6%) | 1,199 (20.1%) |
|  | 12-17y | 285 (15.7%) | 935 (15.7%) |
|  | 18-34y | 263 (14.5%) | 1,321 (22.2%) |
|  | 35-59y | 401 (22.1%) | 1,337 (22.5%) |
|  | 60-89y | 402 (22.1%) | 1,161 (19.5%) |
| **Sex * Age groups** | |  |  |
| *Males* |  | 813 (100%) | 3,260 (100%) |
|  | 0-11y | 234 (28.8%) | 610 (18.7%) |
|  | 12-17y | 140 (17.2%) | 519 (15.9%) |
|  | 18-34y | 91 (11.2%) | 740 (22.7%) |
|  | 35-59y | 160 (19.7%) | 776 (23.8%) |
|  | 60-89y | 188 (23.1%) | 615 (18.9%) |
| *Females* | | 1,002 (100%) | 2,693 (100%) |
|  | 0-11y | 230 (23.0%) | 589 (21.9%) |
|  | 12-17y | 145 (14.5%) | 416 (15.4%) |
|  | 18-34y | 172 (17.2%) | 581 (21.6%) |
|  | 35-59y | 241 (24.1%) | 561 (20.8%) |
|  | 60-89y | 214 (21.4%) | 546 (20.3%) |
| **Country of birth** | | 1,815 (100%) | 5,953 (100%) |
|  | Aruba and (former) Dutch Antilles | 1,170 (64.5%) | 3,627 (60.9%) |
|  | the Netherlands | 249 (13.7%) | 631 (10.6%) |
|  | Other | 396 (21.8%) | 1,695 (28.5%) |
